# Racism in public health services: A research agenda

**DOI:** 10.3389/fpubh.2022.1039963

**Published:** 2022-11-24

**Authors:** Yudit Namer, Lisa Wandschneider, Sigsten Stieglitz, Dagmar Starke, Oliver Razum

**Affiliations:** ^1^Department of Epidemiology and International Public Health, School of Public Health, Bielefeld University, Bielefeld, Germany; ^2^Research Institute Social Cohesion, Bielefeld, Germany; ^3^Akademie für Öffentliches Gesundheitswesen, Düsseldorf, Germany

**Keywords:** racism, public health services, marginalization, stigma, public health authorities

## Abstract

Despite racism and its impact on health inequities being increasingly studied in health care settings, racism in public health services has so far been neglected in public health research. Studying racism in public health services provides many opportunities to explore the relationship between racism and health protection. We identify several research themes to be explored on (1) non-stigmatizing and community-driven risk communication, (2) surveillance by public health authorities of racialized minority groups, (3) racism experiences in everyday interactions with public health authorities, (4) legal consequences of encounters with public health authorities and (5) public health infrastructure, structural racism and the intersectionality of marginalization. Tackling these research themes will help to start building an evidence base on how racism interferes with equitable health protection and how to dismantle it.

## Introduction

Racism is a social and structural determinant of health. Public health work should inherently be anti-racist work, as previous calls for action summarized (1). Despite such calls for action, and despite racism and its impact on health inequities being increasingly studied in health care settings (2), racism in public health services has so far been vastly neglected in public health research.

Structural and institutional racism become the major issues to address in public health services when racialization replaces race as a point of explanation for health protection inequities. Racialization is considered as a society-assigned racial meaning, as opposed to the understanding of race as a biological entity. Racialization can appear permanent and become institutionalized. Stigmatization is considered to be closely linked to racialization: it is one way racialized minority groups are (mis)treated, along with exclusion and punishment (3). Since racism itself racializes and reproduces the different groups, and solidifies hierarchies, it results in health inequities, even when individual racial prejudices are seemingly absent (4).

Concerning public health services, there are groups that might be particularly disadvantaged by inequitable access. These groups range from people seeking care and counseling but also include those who work within public health authorities. Public health authorities have substantial legal powers that can be misused when applied in inequitable or unjust ways. Despite variations by country as well as within countries at the regional or local levels, the roles and responsibilities of public health authorities can manifest as significant control over the lives of individuals and populations often made vulnerable by structural policies. In Germany, where the authors are situated, encounters with public health authorities may carry legal consequences such as being quarantined at home (e.g., in the case of COVID-19), having access to communal housing (e.g., in the case of TB), being able to access abortion, obtaining permit to engage in sex work or being deported based on health certificates provided by public health authorities. Many of these encounters, such as having a viral infection (5), staying in communal housing (6), experiencing an unintended pregnancy (7), engaging in sex work (8), and seeking asylum (9) carry significant stigma. At the same time, these encounters are disproportionately more frequent among multiple stigmatized and socially marginalized groups, including racialized minority groups (3). It is therefore crucial to build an evidence base on whether and how racism interferes with equitable health protection, and how it can be reduced.

Public health authorities are furthermore one of the public offices involved in the surveillance of the population and their health. In this context, racialized data (i.e., data segregated by racial categories in countries where this is done^1^ without addressing structural inequities) play an ambiguous role, most often in relation to infectious diseases. Racialized data are collected, when the country law permits, with the premise of better understanding how infectious diseases affect different communities and helping detect disparities. However, it can become its own form of racialized surveillance, either by complete erasure or misclassification of communities (e.g., Indigenous people) in data collection practices, or by the lack of anonymity of geolocation when communities are segregated (10). Racialized data further generate racialized discourses. Racialized discourses target migration in European settings, especially in Germany (11). For example, despite the fact that migration data were not collected until 2001 in Germany, people coming from “high prevalence countries” were then monitored/screened for HIV in terms of transmission risk until 2011 (12). Besides collecting racialized data, the practice of screening for diseases only in certain racialized minority groups (e.g., refugees for TB, albeit with the motivation to avoid transmission in collective accommodation) additionally contributes to stigmatizing discourses of racialized minority groups (12).

## Research agenda

Studying racism in data collection and population surveillance practices as well as everyday interactions in public health authorities, therefore, provides many opportunities to explore the relationship between racism and health protection. We identify several research themes to be explored:

1) Non-stigmatizing and community-driven risk communication: Effective risk communication entails community-specific health protection and prevention messages, to address special risks and fears. Principles of non-stigmatizing and community-driven communication should be explored to ensure that knowledge from health promotion, prevention and risk communication sciences will be applied appropriately and contradict the implication that disease is inherently tied to racialized identity.
2) Population surveillance and racialized stigma: Surveillance by public health authorities is sometimes intertwined with state surveillance of groups that are racialized. Especially when the cause for being at a higher risk of exposure to health-threatening diseases is structural (e.g., due to refugees being placed in collective accommodation), the screening measures could be taken as a purpose to stigmatize racialized minority groups.
3) Everyday racism and access: Racism experiences of racialized minority groups (including groups seeking health protection and advice, and employees) in their interactions with public health authorities can affect the accessibility of public health services, and trust in institutions in racialized minority groups, exacerbating health inequities.
4) Consequences of public health authority encounters: The (legal) consequences of encounters with public health authorities could create additional disadvantages for people who are already facing intersecting forms of stigmatization and inequalities while the fear of consequences prevents equitable health protection.
5) Historical racism in public health: The public health infrastructure itself is historically deeply intertwined with structural racism and the intersectionality of marginalization (by patriarchy, capitalism and colonialism) which is only beginning to be addressed and explored in public health research (13–15).

## Discussion

Tackling these research themes will help to start building an evidence base on how racism interferes with equitable health protection and how we can dismantle it. All forms of racism in public health authorities, should they indeed exist, ought to be identified and addressed, taking into account statutory and legal contexts that may be structurally racist. Future public health strategy efforts and public health research must build on inclusive and anti-racist approaches. Inclusiveness and anti-racism pertain also to research methods. Public health researchers should aim for participatory and mixed-method studies integrating all stakeholders, in particular staff members of public health authorities and communities affected by racism in public health services. Participatory approaches allow communities to have decision-making power in research that affect their lives and constitute research as intervention (16). Mixed-method approaches allow to include researchers' and participants' perspectives, to explore the experience surrounding research participation, and to understand processes underlying the measured effects (17).

Inclusiveness also relates to the nexus between public health research institutions, public health authorities and training institutions in that public health research should facilitate spaces to enable constructive collaboration and the co-construction of effective interventions. Equally important, public health (research) institutions will have to continue to critically reflect on their institutional practices (e.g., hiring practices and implicit bias) and their positionality within power hierarchies. For that, we can learn from and build partnerships with scholars advancing intersectionality and critical race theory in public health.

## Data availability statement

The original contributions presented in the study are included in the article/supplementary material, further inquiries can be directed to the corresponding author.

## Author contributions

YN prepared the first draft. LW, SS, DS, and OR critically revised the manuscript for important intellectual content. All authors conceptualized the comment, read, and approved the final manuscript.

## Funding

This work has been completed within the Institutions & Racism Consortium, which is funded by the Federal Ministry of the Interior and Community, Germany. We acknowledge support for the publication costs by the Open Access Publication Fund of Bielefeld University and the Deutsche Forschungsgemeinschaft (DFG).

## Conflict of interest

The authors declare that the research was conducted in the absence of any commercial or financial relationships that could be construed as a potential conflict of interest.

## Publisher's note

All claims expressed in this article are solely those of the authors and do not necessarily represent those of their affiliated organizations, or those of the publisher, the editors and the reviewers. Any product that may be evaluated in this article, or claim that may be made by its manufacturer, is not guaranteed or endorsed by the publisher.

## Footnotes

^1^For example, in Germany, in the context of infection protection and the registration of infectious diseases, ethnicity, racial category or nationality is not part of the data set, cf. IfSG (Infection Protection Law) §11.

## References

[1] Jee-Lyn García J Sharif MZ. Black lives matter: A commentary on racism and public health. Am J Public Health. (2015) 105:e27–30. 10.2105/AJPH.2015.302706 26066958 PMC4504294

[2] Ben J Cormack D Harris R Paradies Y. Racism and health service utilisation: A systematic review and meta-analysis. PLoS ONE. (2017) 12:e0189900. 10.1371/journal.pone.0189900 29253855 PMC5734775

[3] Gans HJ. Racialization and racialization research. Ethn Racial Stud. (2017) 40:341–52. 10.1080/01419870.2017.1238497

[4] Namer Y Razum O. ‘Race' causes discomfort? Worse: it misleads. Eur J Public Health. (2021) 31:4–5. 10.1093/eurpub/ckaa198 33253380

[5] Person B Sy F Holton K Govert B Liang A National Center for Inectious Diseases/SARS Community Outreach Team. Fear and stigma: The epidemic within the SARS outbreak. Emerg Infect Dis. (2004) 10:358–63. 10.3201/eid1002.030750 15030713 PMC3322940

[6] Kreichauf R. From forced migration to forced arrival: the campization of refugee accommodation in European cities. CMS. (2018) 6:7. 10.1186/s40878-017-0069-8 29607293 PMC5874268

[7] Kumar A Hessini L Mitchell EMH. Conceptualising abortion stigma. Cult Health Sex. (2009) 11:625–39. 10.1080/13691050902842741 19437175

[8] Lazarus L Deering KN Nabess R Gibson K Tyndall MW Shannon K. Occupational stigma as a primary barrier to health care for street-based sex workers in Canada. Cult Health Sex. (2012) 14:139–50. 10.1080/13691058.2011.628411 22084992 PMC3359131

[9] Satinsky E Fuhr DC Woodward A Sondorp E Roberts B. Mental health care utilisation and access among refugees and asylum seekers in Europe: A systematic review. Health Policy. (2019) 123:851–63. 10.1016/j.healthpol.2019.02.007 30850148

[10] Hendl T Roxanne T. Digital surveillance in a pandemic response: What bioethics ought to learn from Indigenous perspectives. Bioethics. (2022) 36:305–12. 10.1111/bioe.13013 35180324

[11] Wandschneider L Miani C Razum O. Decomposing intersectional inequalities in subjective physical and mental health by sex, gendered practices and immigration status in a representative panel study from Germany. BMC Public Health. (2022) 22:683. 10.1186/s12889-022-13022-1 35392864 PMC8991479

[12] Scott P von Unger H Odukoya D A. tale of two diseases: Discourses on TB, HIV/AIDS and im/migrants and ethnic minorities in the United Kingdom. Soc Theory Health. (2017) 15:261–84. 10.1057/s41285-017-0026-5

[13] Hinz-Wessels A. Das Robert-Koch-Institut im Nationalsozialismus. Berlin: Kulturverlag Kadmos. (2008).

[14] Hulverscheidt M Laukötter A. Infektion und Institution: zur Wissenschaftsgeschichte des Robert Koch-Instituts im Nationalsozialismus. Göttingen: Wallstein (2009).

[15] Donhauser J. “Erb- und Rassenpflege” im Gesundheitsamt: Unterstützung und Ausgrenzung. Gesundheitswesen. (2013) 75:726–9. 10.1055/s-0033-1355367 24142372

[16] von Unger H. Participatory health research with immigrant communities in Germany. Int J Action Res. (2012) 8:266–87. 10.1688/1861-9916_ijar_2012_03_unger29641527

[17] Johnson RB Schoonenboom J. Adding qualitative and mixed methods research to health intervention studies: interacting with differences. Qual Health Res. (2016) 26:587–602. 10.1177/1049732315617479 26657970

